# Supplementary material for: Structural basis of peptide recognition and activation of endothelin receptors
Source: Nat Commun. 2023 Mar 7;14:1268. doi: 10.1038/s41467-023-36998-9 (PMC9992518; doi:10.1038/s41467-023-36998-9)
Supplement: Supplementary file 1 — Supplementary Information [file 41467_2023_36998_MOESM1_ESM.pdf]

Supplementary Information

Structural basis of peptide recognition and activation of endothelin receptors

Yujie Ji[1,2,*], Jia Duan[1,2,*,#], Qingning Yuan[1,*], Xinheng He[1,2], Gong Yang[3], Shengnan Zhu[4], Kai Wu[1], Wen Hu[1], Tianyu Gao[5], Xi Cheng[6], Hualiang Jiang[2,5,6,7], H. Eric Xu[1,2,5,6,#], Yi Jiang[5,7,#]

[1] The CAS Key Laboratory of Receptor Research, Shanghai Institute of Materia Medica, Chinese Academy of Sciences, Shanghai 201203, China

[2] University of Chinese Academy of Sciences, Beijing 100049, China

[3] State Key Laboratory of Cellular Stress Biology, School of Life Sciences, Xiamen University, Fujian 361005, China

[4] School of Pharmacy, Macau University of Science and Technology, Macau 999078, China

[5] School of Life Science and Technology, ShanghaiTech University, Shanghai 201210, China

[6] State Key Laboratory of Drug Research, Shanghai Institute of Materia Medica, Chinese Academy of Sciences, Shanghai 201203, China

[7] Lingang Laboratory, Shanghai 200031, China

[#] Co-corresponding authors:
H.E.X., email: eric.xu@simm.ac.cn,
J. D., email: duanjia@simm.ac.cn
Y. J., email: yjiang@lglab.ac.cn
[*] These authors contributed equally to this work.

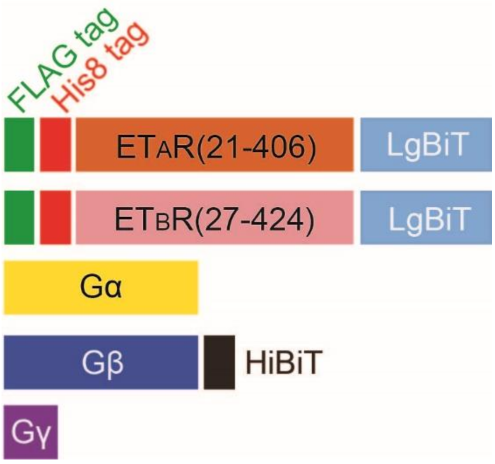

Fig. S1 Engineered constructs used for expression of ET_AR/ET_BR-G protein complexes.

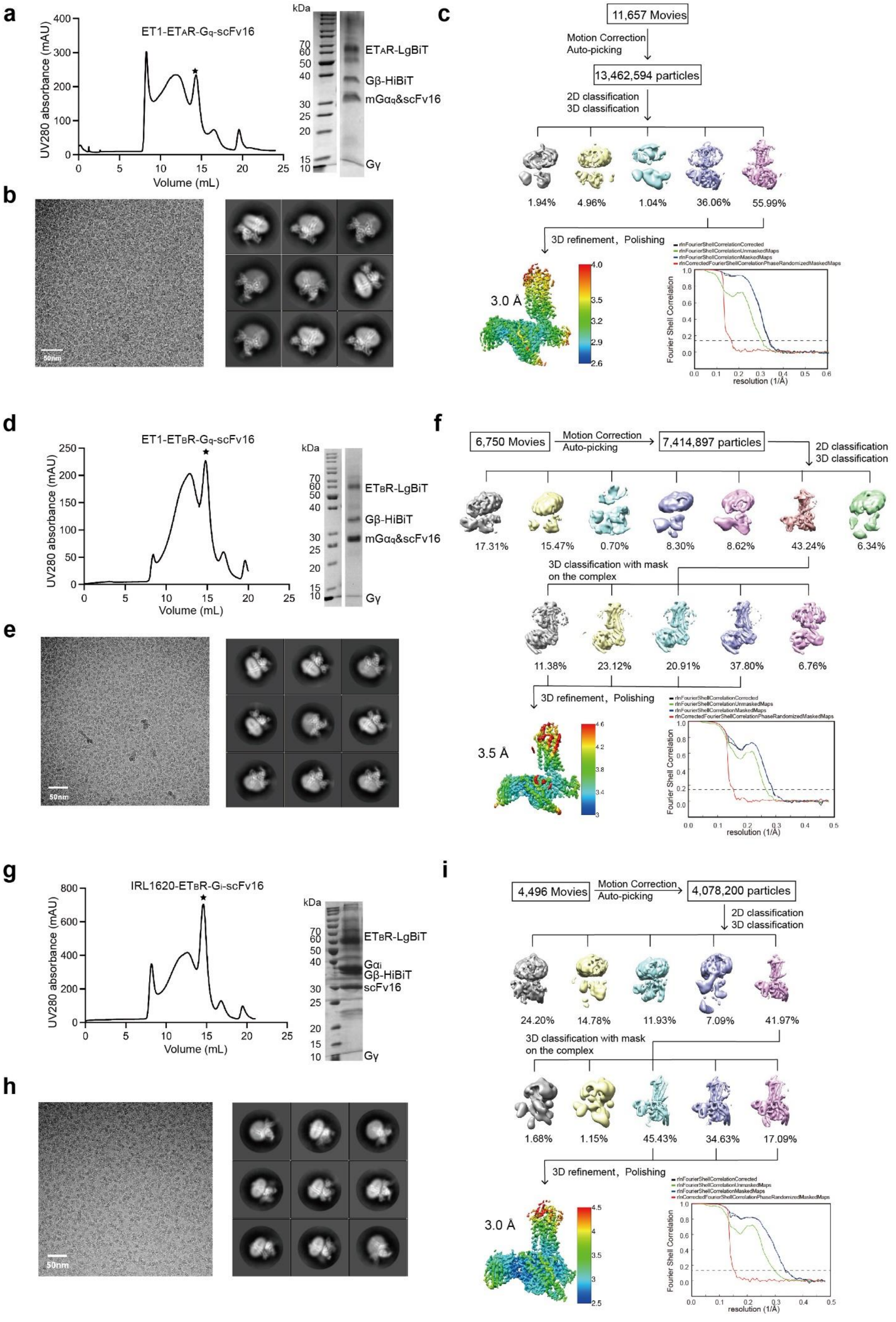
a
ET1-ETAR-Gq-scFv16
UV280 absorbance (mAU)
Volume (mL)
kDa
ETAR-LgBiT
Gβ-HiBiT
mGαq&scFv16
Gγ
b
50nm
c
11,657 Movies
Motion Correction
Auto-picking
13,462,594 particles
2D classification
3D classification
1.94%
4.96%
1.04%
36.06%
55.99%
3D refinement，Polishing
3.0 Å
Fourier Shell Correlation
resolution (1/Å)
d
ET1-ETBR-Gq-scFv16
UV280 absorbance (mAU)
Volume (mL)
kDa
ETBR-LgBiT
Gβ-HiBiT
mGαq&scFv16
Gγ
e
50nm
f
6,750 Movies
Motion Correction
Auto-picking
7,414,897 particles
2D classification
3D classification
17.31%
15.47%
0.70%
8.30%
8.62%
43.24%
6.34%
3D classification with mask
on the complex
11.38%
23.12%
20.91%
37.80%
6.76%
3D refinement，Polishing
3.5 Å
Fourier Shell Correlation
resolution (1/Å)
g
IRL1620-ETBR-Gi-scFv16
UV280 absorbance (mAU)
Volume (mL)
kDa
ETBR-LgBiT
Gαi
Gβ-HiBiT
scFv16
Gγ
h
50nm
i
4,496 Movies
Motion Correction
Auto-picking
4,078,200 particles
2D classification
3D classification
24.20%
14.78%
11.93%
7.09%
41.97%
3D classification with mask
on the complex
1.68%
1.15%
45.43%
34.63%
17.09%
3D refinement，Polishing
3.0 Å
Fourier Shell Correlation
resolution (1/Å)

Fig. S2 The ET-1-ET$_A$R-G$_q$-scFv16, ET-1-ET$_B$R-G$_q$-scFv16, and IRL1620-ET$_B$R-G$_i$-scFv16 complexes purification and cryo-EM data processing. a, d, g Representative elution profile and SDS-PAGE analysis of the ET-1-ET$_A$R-G$_q$-scFv16 (**a**), ET-1-ET$_B$R-G$_q$-scFv16 (**d**) and IRL1620-ET$_B$R-G$_i$-scFv16 complexes (**g**). Black asterisks denote the monomer of three complexes. **b, e, h** Cryo-EM micrographs and reference-free 2D class averages of the ET-1-ET$_A$R-G$_q$-scFv16 (**b**), ET-1-ET$_B$R-G$_q$-scFv16 (**e**) and IRL1620-ET$_B$R-G$_i$-scFv16 complexes (**h**). The complex purification and data collection was performed once. Source data are provided as a Source Data file. **c, f, i** Flow chart of the cryo-EM data processing for the ET-1-ET$_A$R-G$_q$-scFv16 (**c**), ET-1-ET$_B$R-G$_q$-scFv16 (**f**) and IRL1620-ET$_B$R-G$_i$-scFv16 complexes (**i**). The "Gold-standard" Fourier shell correlation (FSC) curve indicates that the overall resolution of the electron density map of the ET-1-ET$_A$R-G$_q$-scFv16 complex is 3.0 Å, the ET-1-ET$_B$R-G$_q$-scFv16 complex is 3.5 Å, and the IRL1620-ET$_B$R-G$_i$-scFv16 complex is 3.0 Å.

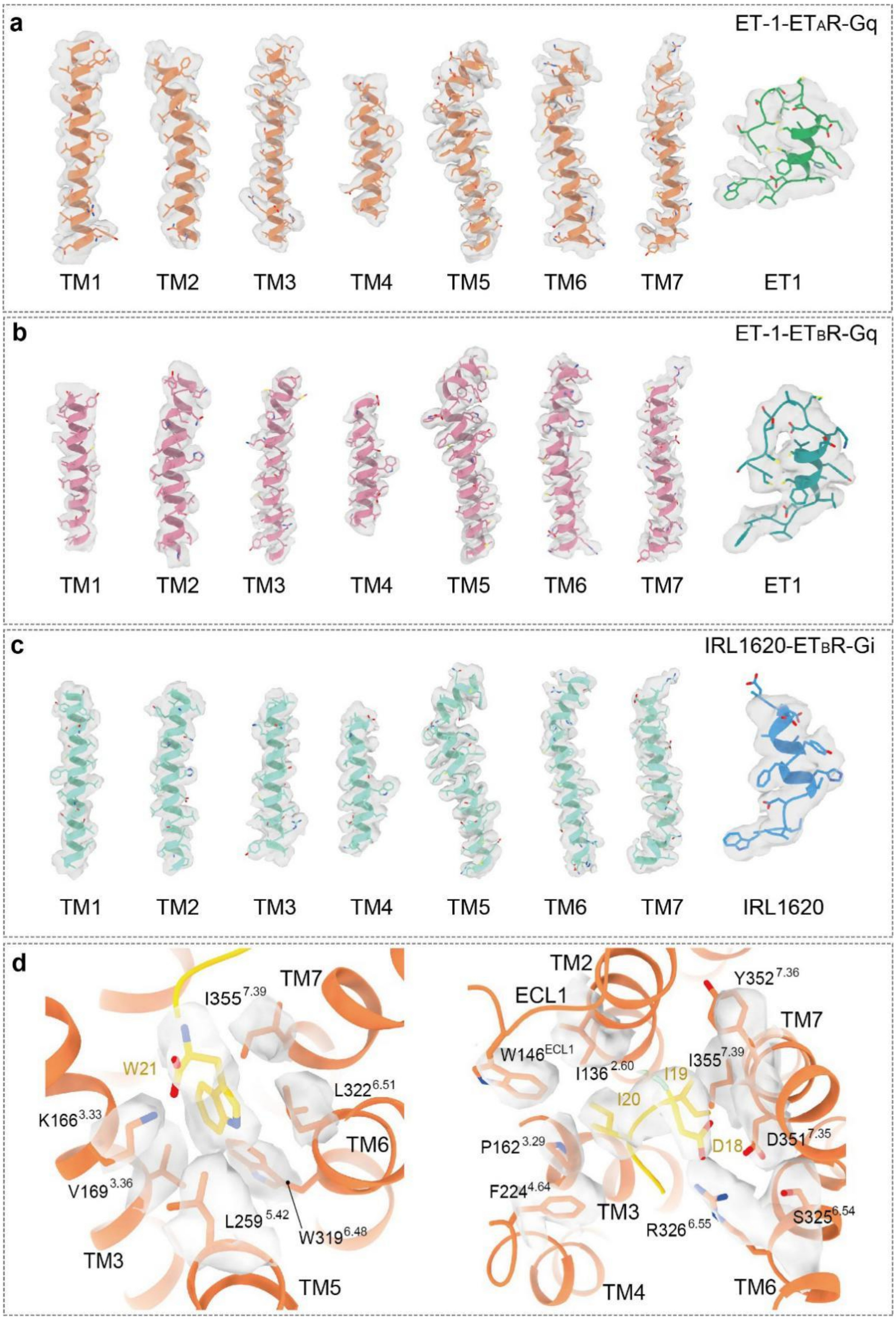

Fig. S3 Representative EM density and coordinate of the ET-1-ET$_A$R-G$_q$-scFv16, ET-1-ET$_B$R-G$_q$-scFv16 and IRL1620-ET$_B$R-G$_i$-scFv16 complexes. EM density and model of ET-1-ET$_A$R-G$_q$ (**a**), ET-1-ET$_B$R-G$_q$ (**b**), IRL1620-ET$_B$R-G$_i$ complexes (**c**), and key ET$_A$R residues in the binding site of the ET-1 C-terminus (**d**).

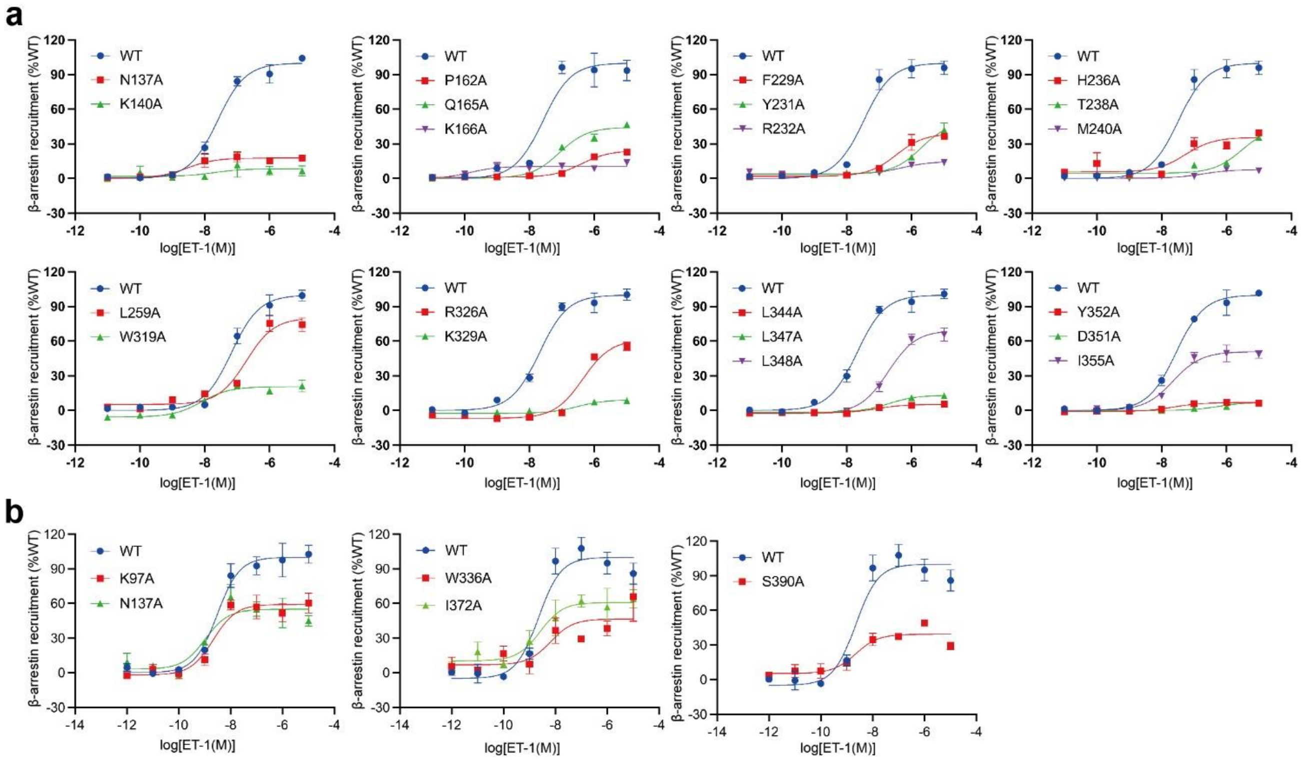

Fig. S4 ET-1 response curves on WT and mutant ET_AR/ET_BR. WT or mutant ET_AR (**a**) or ET_BR (**b**) were transfected into AD293 cells and β-arrestin recruitment signals were measured to reflect the activity of ET-1. The response data was normalized by WT receptor within each individual experiment, with the basal activity for WT as 0, while the fitted E_{max} of WT as 100. Three independent experiments were conducted (n=3). The representative concentration-response curves were shown. Source data are provided as a Source Data file.

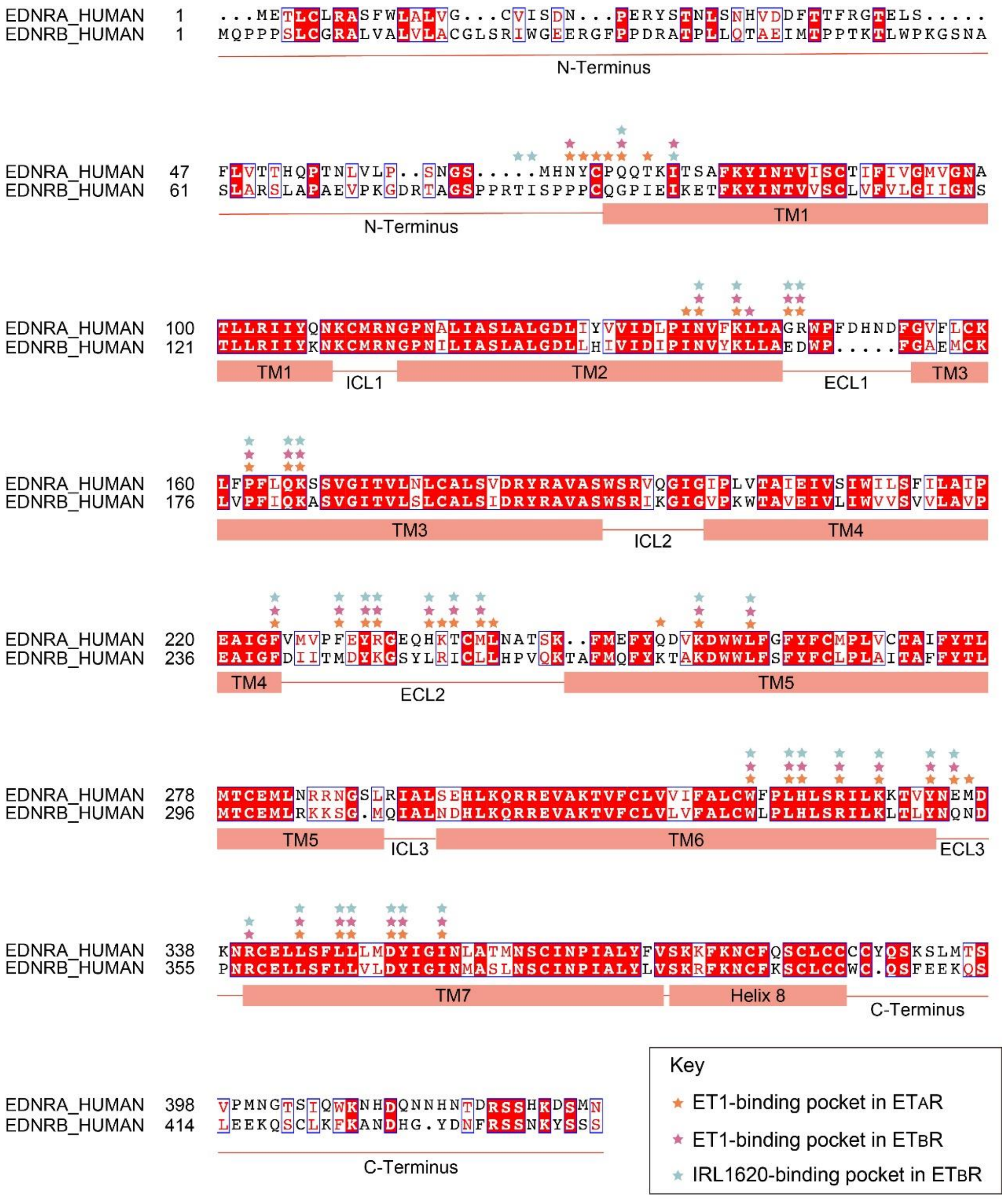

Fig. S5 Sequence alignment of the ETRs. The sequence alignment of ET_AR and ET_BR was generated using NCBI and the graphics was created on the ESPript 3.0 server. α-helices for ETRs are shown as columns underneath the sequences. Orange asterisks represent the binding-pocket residues of ET_AR bound to ET-1, pink asterisks represent the binding-pocket residues of ET_BR bound to ET-1 and cyan asterisks represent the binding-pocket residues of ET_BR bound to IRL1620.

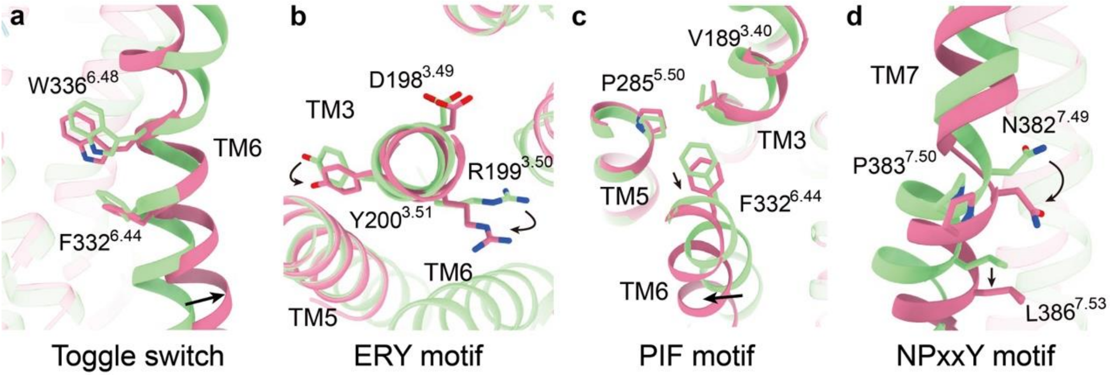

Fig. S6 Conformational changes of the conserved "micro-switches" upon receptor activation. Toggle switch (**a**), ERY motif (**b**), PIF motif (**c**), NPxxY motif (**d**). The outward movement of TM6 of the active receptor is highlighted as a black arrow (**a**). The conformational changes of residue side chains are highlighted as black arrows.

Table S1. Cryo-EM data collection, model refinement, and validation statistics.

	ET-1-ET$_A$R-G$_q$-scFv16 (EMDB-34663) (PDB: 8HCQ)	ET-1-ET$_B$R-G$_q$-scFv16 (EMDB-34667) (PDB: 8HCX)	IRL1620-ET$_B$R-G$_i$-scFv16 (EMDB-34619) (PDB: 8HBD)
**Data collection and processing**			
Magnification	105K	81K	81K
Voltage (kV)	300	300	300
Electron exposure (e–/Å^2)	50	50	50
Defocus range (μm)	-0.8 ~ -1.8	-0.8 ~ -2	-0.8 ~ -2
Pixel size (Å)	0.824	1.04	1.04
Symmetry imposed	C1	C1	C1
Initial particle images (no.)	13,462,594	7,414,897	4,078,200
Final particle images (no.)	510,197	373,655	191,493
Map resolution (Å)	3.0	3.5	3.0
FSC threshold	0.143	0.143	0.142
Map resolution range (Å)	2.6-4.0	3.0-4.6	2.5-4.5
**Refinement**			
Initial model used (PDB code)	None	None	None
Model resolution (Å)	3.4	4	3.4
FSC threshold	0.5	0.5	0.5
Map sharpening *B* factor (Å^2)	-106.707	-131.676	-90.3371
Model composition			
Non-hydrogen atoms	9013	8933	8826
Protein residues	1157	1159	1158
Ligands	0	0	0
B factors (Å^2)			
Protein	102.61	95.47	83.46
Ligand	0	0	0
R.m.s. deviations			
Bond lengths (Å)	0.003	0.003	0.002
Bond angles (°)	0.661	0.623	0.575
Validation			
MolProbity score	1.75	1.7	1.71
Clashscore	9.15	10.83	10.70
Poor rotamers (%)	0.21	0.31	0.32
Ramachandran plot			
Favored (%)	96.11	97.19	97.08
Allowed (%)	3.8	2.46	2.83
Disallowed (%)	0.09	0.35	0.09

Table S2. Effects of ET-1 on ET_AR/ET_BR mutants. NanoBiT assay was performed to evaluate the effects of ET1 on β-arrestin 2 of ET_AR/ET_BR. The surface expression of each ET_AR/ET_BR mutant was normalized to wild-type (WT) receptor, which was set to 100%. Data are presented as means ± S.E.M. of three independent experiments in triplicate (n = 3). U.D., undetectable. Source data are provided as a Source Data file.

Receptor	Residue number	Mutant	EC_{50} (nM)	Fold of change	Surface expression(%WT)
ET_AR	-	WT	20.71 ± 0.56	1	100
	2.61	N137A	U.D.	-	87.85 ± 2.65
	2.64	K140A	U.D.	-	88.94 ± 6.76
	3.29	P162A	>376.50	>18.18	97.65 ± 6.94
	3.32	Q165A	> 161.07	>7.78	63 ± 1.3
	3.33	K166A	U.D.	-	126.09 ± 7.12
	ECL2	F229A	>657.60	>31.75	105.28 ± 7.64
	ECL2	Y231A	U.D.	-	64.39 ± 7.04
	ECL2	R232A	U.D.	-	80.72 ± 8.26
	ECL2	H236A	60.92 ± 8.39	2.94	91.01 ± 8.39
	ECL2	T238A	U.D.	-	55.85 ± 5.13
	ECL2	M240A	U.D.	-	72.84 ± 4.46
	5.42	L259A	190.73 ± 11.26	9.21	60.26 ± 9.19
	6.48	W319A	5.89 ± 0.83	0.28	108.92 ± 2.68
	6.55	R326A	>424.17	>20.48	60.86 ± 8.38
	6.58	K329A	U.D.	-	88.41 ± 5.28
	7.28	L344A	127.96 ± 17.23	6.18	100.89 ± 8.23
	7.31	L347A	U.D.	-	69.16 ± 3.76
	7.32	L348A	U.D.	-	70.72 ± 0.43
	7.35	D351A	U.D.	-	106.9 ± 2.04
	7.36	Y352A	U.D.	-	80.24 ± 6.83
	7.39	I355A	U.D.	-	54.53 ± 6.4
ET_BR	-	WT	2.50 ± 0.20	1	100
	1.28	K97A	1.68 ± 0.33	0.67	85.63 ± 2.04
	2.40	N137A	2.79 ± 0.88	1.12	50.48 ± 4.73
	6.48	W336A	5.55 ± 0.58	2.22	71.82 ± 4.97
	7.39	I372A	2.12 ± 0.34	0.85	111.70 ± 5.32
	8.47	S390A	2.57 ± 0.48	1.03	110.75 ± 3.33

Table S3. List of primers sequences for site-direct mutagenesis studies.

Receptor	primers	Forward	Reverse
**ET_AR**	$N137^{2.61}A$	CTATCGCTGTATTTAAGCTGCTG	AATACAGCGATAGGGAGATCAAT
	$K140^{2.64}A$	TATTTGCTCTGCTGGCTGGGCGC	AGCAGAGCAAATACATTGATAGG
	$P162^{3.29}A$	TGTTCGCTTTTTTGCAGAAGTCCT	AAAAAAGCGAACAGCTTGCAAAG
	$Q165^{3.32}A$	TTTTGGCTAAGTCCTCGGTGGGG	GACTTAGCCAAAAAGGGGAACAG
	$K166^{3.33}A$	TGCAGGCTTCCTCGGTGGGGATC	GAGGAAGCCTGCAAAAAGGGGAA
	$F229^{ECL2}A$	TACCCGCTGAATATAGGGGTGAA	TATTCAGCGGGTACCATGACGAA
	$Y231^{ECL2}A$	TTGAAGCTAGGGGTGAACAGCAT	CCCCTAGCTTCAAAGGGTACCAT
	$R232^{ECL2}A$	AATATGCTGGTGAACAGCATAAA	TCACCAGCATATTCAAAGGGTAC
	$H236^{ECL2}A$	AACAGGCTAAAACCTGTATGCTC	GTTTTAGCCTGTTCACCCCTATA
	$T238^{ECL2}A$	ATAAAGCTTGTATGCTCAATGCC	ATACAAGCTTTATGCTGTTCACC
	$M240^{ECL2}A$	CCTGTGCTCTCAATGCCACATCA	TTGAGAGCACAGGTTTTATGCTG
	$L259^{5.42}A$	GGTGGGCTTTCGGGTTCTATTTC	CCGAAAGCCCACCAGTCCTTTAC
	$W319^{6.48}A$	TTTGCGCTTTCCCTCTTCATTTA	GGGAAAGCGCAAAGAGCAAAAAT
	$R326^{6.55}A$	TAAGCGCTATATTGAAGAAAACT	AATATAGCGCTTAAATGAAGAGG
	$K329^{6.58}A$	TATTGGCTAAAACTGTGTATAAC	GTTTTAGCCAATATACGGCTTAA
	$L344^{7.28}A$	AATTAGCTAGTTTCTTACTGCTC	AAACTAGCTAATTCACATCGGTT
	$L347^{7.31}A$	GTTTCGCTCTGCTCATGGATTAC	AGCAGAGCGAAACTAAGTAATTC
	$L348^{7.32}A$	TCTTAGCTCTCATGGATTACATC	ATGAGAGCTAAGAAACTAAGTAA
	$D351^{7.35}A$	TCATGGCTTACATCGGTATTAAC	ATGTAAGCCATGAGCAGTAAGAA
	$Y352^{7.36}A$	TGGATGCTATCGGTATTAACTTG	CCGATAGCATCCATGAGCAGTAA
	$I355^{7.39}A$	TCGGTGCTAACTTGGCAACCATG	AAGTTAGCACCGATGTAATCCAT
**ET_BR**	$K97^{1.28}A$	AAATCGCAGAGACCTTCAAGTAC	AGGTCTCTGCGATTTCGATAGGACC
	$N137^{2.40}A$	GTCCTGCTATCCTGATCGCTTCTTTG	TCAGGATAGCAGGACCGTTACGCATG
	$W336^{6.48}A$	TCTGTGCTTTGCCTTTGCACTTG	AAGGCAAAGCACAGAGAGCGAACAC
	$I372^{7.39}A$	TCGGAGCTAACATGGCTTCCCTG	CCATGTTAGCTCCGATGTAGTCCAG
	$S390^{8.47}A$	TGGTGGCTAAGCGCTTCAAGAATTG	AGCGCTTAGCCACCAGGTACAAAGC
