# Supplementary material for: Structural basis of peptide recognition and activation of endothelin receptors
Source: Nat Commun. 2023 Mar 7;14:1268. doi: 10.1038/s41467-023-36998-9 (PMC9992518; doi:10.1038/s41467-023-36998-9)
Supplement: Supplementary file 2 — Reporting Summary [file 41467_2023_36998_MOESM2_ESM.pdf]

nature portfolio

Corresponding author(s): Yi Jiang
H.Eric Xu

Last updated by author(s): Feb 3, 2023

Reporting Summary

Nature Portfolio wishes to improve the reproducibility of the work that we publish. This form provides structure for consistency and transparency in reporting. For further information on Nature Portfolio policies, see our Editorial Policies and the Editorial Policy Checklist.

Statistics

For all statistical analyses, confirm that the following items are present in the figure legend, table legend, main text, or Methods section.

n/a	Confirmed	
☐	☒	The exact sample size (*n*) for each experimental group/condition, given as a discrete number and unit of measurement
☐	☒	A statement on whether measurements were taken from distinct samples or whether the same sample was measured repeatedly
☒	☐	The statistical test(s) used AND whether they are one- or two-sided<br>*Only common tests should be described solely by name; describe more complex techniques in the Methods section.*
☒	☐	A description of all covariates tested
☒	☐	A description of any assumptions or corrections, such as tests of normality and adjustment for multiple comparisons
☐	☒	A full description of the statistical parameters including central tendency (e.g. means) or other basic estimates (e.g. regression coefficient) AND variation (e.g. standard deviation) or associated estimates of uncertainty (e.g. confidence intervals)
☒	☐	For null hypothesis testing, the test statistic (e.g. *F*, *t*, *r*) with confidence intervals, effect sizes, degrees of freedom and *P* value noted<br>*Give P values as exact values whenever suitable.*
☒	☐	For Bayesian analysis, information on the choice of priors and Markov chain Monte Carlo settings
☒	☐	For hierarchical and complex designs, identification of the appropriate level for tests and full reporting of outcomes
☒	☐	Estimates of effect sizes (e.g. Cohen's *d*, Pearson's *r*), indicating how they were calculated

Our web collection on statistics for biologists contains articles on many of the points above.

Software and code

Policy information about availability of computer code

Data collection	Atuomated data collection on the Titan Krios G4 using EPU 2.11.
Data analysis	RELION-4.0-beta, CTFFIND 4.0, Alphafold2, UCSF Chimera 1.15, UCSF Chimera X 1.2.5, Phenix 1.20.1, Coot 0.9.6, PyMOL 2.5.2, Prism 9, Accuri C6 software1.0.264.21

For manuscripts utilizing custom algorithms or software that are central to the research but not yet described in published literature, software must be made available to editors and reviewers. We strongly encourage code deposition in a community repository (e.g. GitHub). See the Nature Portfolio guidelines for submitting code & software for further information.

Data

Policy information about availability of data

All manuscripts must include a data availability statement. This statement should provide the following information, where applicable:

- Accession codes, unique identifiers, or web links for publicly available datasets
- A description of any restrictions on data availability
- For clinical datasets or third party data, please ensure that the statement adheres to our policy

Density maps and structure coordinates have been deposited in the Electron Microscopy Data Bank (EMDB) and the Protein Data Bank (PDB) with accession codes EMD-34663 and 8HCQ for the ET-1-ETAR-Gq-scFv16 complex; EMD-34667 and 8HCX for the ET-1-ETBR-Gq-scFv16 complex; EMD-34619 and 8HBD for the IRL1620-ETBR-Gi-scFv16 complex.

Human research participants

Policy information about studies involving human research participants and Sex and Gender in Research.

Reporting on sex and gender	N/A
Population characteristics	N/A
Recruitment	N/A
Ethics oversight	N/A

Note that full information on the approval of the study protocol must also be provided in the manuscript.

Field-specific reporting

Please select the one below that is the best fit for your research. If you are not sure, read the appropriate sections before making your selection.

☒ Life sciences ☐ Behavioural & social sciences ☐ Ecological, evolutionary & environmental sciences

For a reference copy of the document with all sections, see nature.com/documents/nr-reporting-summary-flat.pdf

Life sciences study design

All studies must disclose on these points even when the disclosure is negative.

Sample size	Sample sizes were not predetermined by statistical methods. For cryo-EM data, images were collected until the resolution and 3D reconstruction converges. For all the functional assay, at least three independent experiments were conducted. The sample size is sufficient for statistical analysis.
Data exclusions	No data were excluded from the analysis.
Replication	All the functional assay were conducted at least three independent experiments with technical repeats. All attempts at replication were successful.
Randomization	Randomization was not relevant to this study, as the data were collected automatically and did not involve choosing.
Blinding	Blinding was not required for the structural and functional experiments because no subjective assignment was involved.

Reporting for specific materials, systems and methods

We require information from authors about some types of materials, experimental systems and methods used in many studies. Here, indicate whether each material, system or method listed is relevant to your study. If you are not sure if a list item applies to your research, read the appropriate section before selecting a response.

Materials & experimental systems

n/a	Involved in the study	
☐	☒	Antibodies
☐	☒	Eukaryotic cell lines
☒	☐	Palaeontology and archaeology
☒	☐	Animals and other organisms
☒	☐	Clinical data
☒	☐	Dual use research of concern

Methods

n/a	Involved in the study	
☒	☐	ChIP-seq
☒	☐	Flow cytometry
☒	☐	MRI-based neuroimaging

Antibodies

Antibodies used	Monoclonal ANTI-FLAG M2 antibody (Sigma-Aldrich, F3165);<br>Goat anti-Mouse IgG (H+L) Highly Cross-Adsorbed Secondary Antibody, Alexa Fluor 488 (ThermoFisher, A-11029)
Validation	All antibodies used were commercially available and validated by the manufacturer for use as described in this study.<br>Monoclonal ANTI-FLAG M2 antibody (https://www.sigmaaldrich.com/HK/en/product/sigma/f3165);

Goat anti-Mouse IgG (H+L) Highly Cross-Adsorbed Secondary Antibody, Alexa Fluor 488 (https://www.thermofisher.cn/cn/zh/antibody/product/Goat-anti-Mouse-IgG-H-L-Highly-Cross-Adsorbed-Secondary-Antibody-Polyclonal/A-11029)

Eukaryotic cell lines

Policy information about cell lines and Sex and Gender in Research

Cell line source(s)	Sf9 (Expression Systems, Cat#94-001F)<br>AD293 (Agilent)
Authentication	Used as expression stains only, independent verification after purchase not required.
Mycoplasma contamination	Cell lines were tested and free from mycoplasma contamination.
Commonly misidentified lines<br>(See ICLAC register)	No commonly misidentified cell lines were used.
